# Obstructive sleep apnea and nocturnal hypoxemia in adult patients with cystic fibrosis

**DOI:** 10.1186/s12890-022-02243-0

**Published:** 2022-11-28

**Authors:** Matthias Welsner, Sarah Dietz-Terjung, Florian Stehling, Tim Schulte, Ute Niehammer, Fatma-Ezzahra Gahbiche, Christian Taube, Svenja Strassburg, Christoph Schoebel, Gerhard Weinreich, Sivagurunathan Sutharsan

**Affiliations:** 1 Department of Pulmonary Medicine, Adult Cystic Fibrosis Center, University Hospital Essen - Ruhrlandklinik, University of Duisburg-Essen, Essen, Germany; 2 Department of Sleep and Telemedicine, University Hospital Essen - Ruhrlandklinik, University of Duisburg-Essen, Essen, Germany; 3 Pediatric Pulmonology and Sleep Medicine, Cystic Fibrosis Center, Children´s Hospital, University of Duisburg-Essen, Essen, Germany

**Keywords:** Cystic fibrosis, Adults, Polysomnography, Excessive daytime sleepiness, Apnea-hypopnea index, Obstructive sleep apnea, Nocturnal hypoxemia

## Abstract

**Background:**

Obstructive sleep apnea (OSA), nocturnal hypoxemia and excessive daytime sleepiness (EDS) are common comorbidities in people with cystic fibrosis (pwCF). Most of the data showing this originates from children and adolescents. The aim of this study was to collect data on sleep parameters, EDS and pulmonary function from a large cohort of adult pwCF.

**Methods:**

Full overnight polysomnography (PSG) was performed. EDS was determined using the Epworth Sleepiness Scale (ESS). Demographic and clinical data (body mass index [BMI], pulmonary function, capillary blood gases) were collected.

**Results:**

A total of 52 adult pwCF were included (mean age 30.7 ± 8.0 years, mean percent predicted forced expiratory volume in 1 s [ppFEV_1_] of 52.1 ± 14.8). Overall AHI was in the normal range (4.5 ± 4.0/h); 21/52 pwCF (40%) had an apnea-hypopnea index > 5/h. Nocturnal hypoxemia was found in 25% of participants and this was associated with ppFEV_1_ (*p* = 0.014), awake oxygen saturation (SpO_2_; *p* = 0.021) and awake partial pressure of oxygen (pO_2_; *p* = 0.003); there were no significant differences in age, lung function and BMI were found for pwCF with versus without OSA (all *p* > 0.05). Eight pwCF (15%) had an ESS score > 10 (indicating EDS). OSA was best predicted by awake pO_2_ (area under the curve [AUC] 0.66, *p* = 0.048), while nocturnal hypoxemia was best predicted by ppFEV_1_ (AUC 0.74, *p* = 0.009), awake pO_2_ (AUC 0.76, *p* = 0.006) and awake SpO_2_ (AUC 0.71; *p* = 0.025).

**Conclusion:**

OSA, nocturnal hypoxemia and EDS were common in adult pwCF, but no strong predictors were identified. Therefore, we suggest regular PSG and ESS scoring in adult pwCF, regardless of disease severity.

## Background

Cystic fibrosis (CF), an autosomal recessive monogenetic disorder, is caused by mutations in the CF transmembrane conductance regulator (CFTR) gene on chromosome 7 [1]. This results in disturbed anion transport (Cl^−^ and HCO_3_^−^) through epithelial cell membranes and therefore to the formation of highly viscous secretions in all exocrine organs. The organs that are primarily affected are the lungs and the gastro-intestinal tract, leading to progressive lung damage and malnutrition [2]. Mortality and morbidity are mainly caused by lung involvement with progressive obstructive lung disease, hyperinflation, impaired gas exchange and end-stage respiratory failure [3]. Due to advances in the treatment of people with cystic fibrosis (pwCF), life expectancy has increased to more than 50 years of age and the number of adults with CF now exceeds the number of children with the disease [4].

As with other chronic obstructive lung diseases, such as chronic obstructive pulmonary disease (COPD) and asthma [5, 6], pwCF of all ages may also have sleep-disordered breathing (SDB), mainly obstructive sleep apnea (OSA) and nocturnal hypoxemia [7–9]. The gold standard technique for diagnosing SDB is overnight, in-laboratory polysomnography (PSG), as recommended by the American Academy of Sleep Medicine (AASM) [10]. An apnea-hypopnea index (AHI) of ≥ 5/h is required for diagnosis of OSA. The prevalence of OSA in the general population is up to 38%, with (overweight) males and elderly people most likely to be affected [11]. The reported prevalence of OSA in pwCF varies widely, being as high as 70% in children [12] and up to 3.9% in adults [13].

Nocturnal hypoxemia is also a common finding in pwCF [14–16]. Sleep studies in pwCF of all ages showed a direct correlation between the presence of nocturnal hypoxemia and lung disease severity [13, 15, 17]. In addition, nocturnal hypoxemia may be present even in children with mild lung disease [12]. Furthermore, hypoventilation with hypercapnia requiring non-invasive ventilation is common in individuals with advanced CF [18, 19].

Poor subjective and objective sleep quality is a well-known comorbidity in pwCF across all age groups [20–22]. In addition to disease-specific causes of disturbed sleep, such as coughing or nocturnal PEG feeding, SDB is also likely to play an important role. Impaired sleep and SDB may have a variety of consequences in pwCF, including reduced health-related quality of life (HRQoL) [20, 23], increased daytime sleepiness [20, 24], reduced neurocognitive function [24], development of pulmonary hypertension [25], or reduced physical activity [26]. Despite the knowledge that SDB is a common comorbidity in pwCF and its negative impact on different aspects of the disease, most CF centers do not screen routinely for SDB [27]. Recent reviews highlighted the significant impact of SDB for pwCF. Further research with larger sample sizes was requested to better define SDB in pwCF and to avoid nonattention of this relevant comorbitiy.

The aims of this study were to determine the presence of OSA and nocturnal hypoxemia in a large cohort of adult pwCF, to assess the clinical characteristics of pwCF with and without OSA, nocturnal hypoxemia and excessive daytime sleepiness (EDS), and to evaluate possible clinical parameters for predicting OSA and nocturnal hypoxemia in pwCF.

### Methods

#### Study design

This prospective, observational, and descriptive-analytical study included adult pwCF from the Adult Cystic Fibrosis Unit of the Ruhrlandklinik Essen, Germany, who were recruited between September and December 2020. The study was approved by the local ethics committee of the University Hospital Essen (19-8961-BO) and followed the Declaration of Helsinki Ethical Principles for Medical Research Involving Human Subjects. All pwCF provided written informed consent for participation in the study.

#### Participants

All participants were ≥ 18 years of age and had a diagnosis of CF based on the presence of two defining mutations in the CFTR gene. All participants were clinically stable without signs of respiratory exacerbation, on stable medication and had a stable percent predicted forced expiratory volume in 1 s (ppFEV_1_) for a minimum of 4 weeks prior to the study assessments.

#### Polysomnography and Epworth Sleepiness Scale (ESS)

In-lab PSG was performed using a digital polygraph (Nox Medical, Iceland). Two electroencephalograms (EEG), two electrooculograms (EOG), submental and tibialis electromyogram (EMG), rib cage and abdominal inductance pneumograms, pulse oximeter (Nonin, Minnesota, USA), nasal cannula (measurement of flow at a sample frequency of 20 Hz), and body position were recorded. Using the AASM standard [28], an apnea was defined as a flow cessation for ≥ 10 s, and a hypopnea was defined as a ≥ 50% reduction in flow amplitude or a ≥ 30% decrease in flow amplitude accompanied by a > 3% reduction in oxygen saturation lasting ≥ 10 s. Relevant oxygen desaturation during sleep was defined as an oxygen saturation (SpO_2_) of < 90% for ≥ 5% of total sleep time (TST) with a nadir of at least 85%, as described elsewhere [16].

All signals were recorded automatically and subsequently analyzed blinded by the same German Sleep Society (DGSM) trained investigator (SDT) to prevent inter-rater variability. The AHI was defined as the number of apneas and hypopneas per hour of sleep. Depending on whether they occurred in rapid eye movement (REM) or non-REM (NREM) sleep phases, AHI was further classified as AHI REM or AHI NREM.

After diagnostic PSG, participants were asked to answer the ESS questionnaire, a self-report instrument that addresses the possibility of falling asleep in daily life. The score consists of 8 items (0–3 points each) ranging from 0 to 24. A score of > 10 indicates daytime sleepiness [29].

#### Pulmonary function testing (PFT) and body mass index

Calculation of the body mass index (BMI) and pulmonary function testing (PFT) were performed on the day of, or one day after, the diagnostic sleep study. Forced vital capacity (FVC), forced expiratory volume in 1 s (FEV_1_) and residual volume (RV) were measured with a JAEGER MasterScreen Body (CareFusion, Hoechberg, Germany) according to ATS guidelines [30]. Global Lung Function Initiative reference values were used [31]. Arterialized ear lobe blood gas samplings were used to assess pH, partial pressure of oxygen (pO_2_), partial pressure of carbon dioxide (pCO_2_), base excess (BE), and bicarbonate (HCO_3_^-^). This analysis was performed before PFT.

### Statistical analysis

Statistical analysis was performed using the SPSS statistics package version 27 (SPSS Inc., Chicago, USA). Data are presented as mean ± standard deviation. The Shapiro-Wilk test was used to evaluate the data for normal distribution. Student’s t-test or Mann-Whitney U-test were used to assess between-group differences, as appropriate. A p-value of < 0.05 was considered statistically significant.

Receiver operator characteristics (ROC) analysis was performed to identify predictors of OSA and nocturnal hypoxemia using ppFEV_1_, BMI, awake SpO_2_ and pO_2_, ESS score and age as variables. GraphPad Prism version 9.3 (GraphPad Software, San Diego, USA) was used for plotting ROC curves and ROC analysis, including determination of the area under the ROC curve (AUC), specificity and sensitivity for the single variables.

## Results

### Study population

A total of 64 pwCF were recruited, all of whom underwent full PSG. Twelve patients were excluded from statistical analysis due to having a TST < 180 min or an insufficient sleep data quality or missing PFT data. The remaining 52 pwCF were included in the analysis (Table 1). Two patients used nocturnal oxygen supplementation, which was paused during the diagnostic night. No patient was using nocturnal continuous or bilevel positive airway pressure (CPAP/BiPAP) therapy.

**Table 1:** Patient clinical and demographic characteristics

Characteristics	Patients (*n* = 52)
Age, years	30.7 ± 8.0 (20–49)
Female, n (%)	18 (35)
Genotype, n (%)	
F508del homozygous	38 (73)
F508del heterozygous	14 (27)
CFTR modulator therapy, n (%)	
None	16 (31)
Tezacaftor/ivacaftor	33 (63)
Lumacaftor/ivacaftor	3 (6)
Body mass index, kg/m^2^	21.5 ± 3.3 (15.6–31.2)
FEV_1_, L	2.1 ± 0.8 (1.0-4.8)
FEV_1_, % predicted	52.1 ± 14.8 (30.0–96.0)
FVC, L	3.4 ± 1.1 (1.5–6.2)
FVC, % predicted	69.5 ± 16.2 (37.0-105.0)
Residual volume, L	3.0 ± 0.9 (1.1–5.6)
Residual volume, % predicted	179.9 ± 41.9 (99.0-283.0)
Pancreatic insufficiency, n (%)	51 (98)
*Pseudomonas aeruginosa* positive, n (%)	28 (54)
Cystic fibrosis-related diabetes, n (%)	12 (23)
Oxygen supplementation, n (%)	2 (4)

Data are mean ± standard deviation (range) or number of patients (%)

CFTR, cystic fibrosis transmembrane conductance regulator; FEV_1_, forced expiratory volume in 1 s; FVC, forced vital capacity

Mean age of the study population was 30.7 ± 8.0 years with a mean ppFEV_1_ of 52.1 ± 14.8 (Table 1). BMI ranged from 15.6 to 31.2 kg/m^2^ (mean 21.5 ± 3.3) and 15% of patients had a BMI > 25 kg/m^2^ (Table 1). The ESS score was 6.7 ± 3.8 overall, and 15% of patients had an ESS score of > 10 (Table 1).

#### Respiratory events and nocturnal gas exchange

Overall, the AHI was in the normal range (4.5 ± 4.0 events/h), and was higher during REM sleep (Table 2). Awake capillary blood gas analysis revealed normal values. Mean and minimum nocturnal SpO_2_ values were 92.1 ± 2.2% and 87.0 ± 3.8% respectively (Table 2). Thirteen patients (25%) had significant nocturnal hypoxemia (SpO_2_ < 90% for more than 5% of TST and a nadir of at least 85%), and mean sleep time spent with SpO_2_ < 90% was 41.3 min (maximum 321.3 min) (Table 2).

**Table 2:** Polysomnographic data

	Patients (*n* = 52)
AHI, events/h	4.5 ± 4.0 (0-15.5)
AHI > 5 events/h, n (%)	21 (40)
AHI REM, events/h	10.2 ± 10.2 (0-38.5)
AHI NREM, events/h	3.1 ± 3.4 (0-17.3)
Arousal index, events/h	18.8 ± 10.2 (0.5–46.4)
ESS score	6.7 ± 3.8 (0–22)
ESS score > 10, n (%)	8 (15)
TST, min	298.1 ± 43.8 (180–400)
Sleep efficiency, %	74.9 ± 10.2 (48.6–93.9)
Sleep latency, min	69.6 ± 37.4 (8.8-151.2)
WASO, min	33.3 ± 27.7 (0.5-145.5)
Sleep stages, % TST	
N1	3.1 ± 1.8 (0.5-9.0)
N2	51.0 ± 7.9 (31.3–68.7)
N3	25.5 ± 8.9 (11.1–49.8)
REM	17.7 ± 6.6 (5.2–34.3)
ODI, events/h	4.2 ± 3.8 (0-15.5)
ODI REM, events/h	11.2 ± 13.8 (0–80.0)
ODI NREM, events/h	2.9 ± 3.1 (0-15.7)
Nocturnal mean SpO_2_, %	92.1 ± 2.2 (84–96)
Nocturnal minimum SpO_2_, %	87.0 ± 3.8 (77–92)
SpO_2_ < 90%, % TST	13.4 ± 27.3 (0.0-99.9)
SpO_2_ < 90%, min	41.3 ± 85.5 (0.0-321.3)
Nocturnal respiratory rate, breaths/min	21.4 ± 3.9 (15.0-31.2)
Nocturnal heart rate, beats/min	67.9 ± 12.5 (49.9-102.3)
Awake SpO_2_, %	95.8 ± 1.7 (91.0–98.0)
Awake pO_2_, mmHg	78.6 ± 9.4 (57.0–97.0)
Awake pCO_2_, mmHg	38.9 ± 2.9 (33.0–45.0)
Awake pH	7.4 ± 0.3 (7.29–7.50)
Awake HCO_3_^−^, mmol/L	24.9 ± 2.0 (19.1–30.5)

Values are mean ± standard deviation (range) or number of patients (%)

AHI, apnea-hypopnea index; ESS, Epworth Sleepiness Scale; HCO_3_^−^, bicarbonate; NREM, non-rapid eye movement sleep; ODI, oxygen desaturation index; pCO_2_, partial pressure of carbon dioxide pressure; pO_2_, partial pressure of oxygen; REM, rapid eye movement sleep; SpO_2_, oxygen saturation; TST, total sleep time; WASO, wake after sleep onset

#### Sleep structure

Adult pwCF showed decreased sleep efficiency 74.9 ± 10.2%) and increased sleep latency (69.6 ± 37.4 min) (Table 2). Sleep architecture was in the normal range for time spent in N1-3 and REM sleep (Table 2).

#### Profiles of pwCF with and without OSA

Twenty-one of 52 pwCF (40%) fulfilled polysomnographic criteria for OSA (AHI > 5/h). There were no significant differences between pwCF with and without OSA with respect to age, lung function, BMI, ESS score, sleep architecture and sleep quality (Table 3). Those with versus without OSA had significantly lower nocturnal oxygen levels (Table 3). Respiratory rates did not differ between the two groups, whereas pwCF with OSA had a slightly higher nocturnal heart rate than those without OSA (p < 0.049) (Table 3). Only two of the 21 pwCF with OSA has an ESS score > 10 (Table 3).

**Table 3:** Comparison of clinical characteristics and polysomnographic data in people with cystic fibrosis with and without obstructive sleep apnea

	OSA (*n* = 21)	No OSA (*n* = 31)	p value
Age, years	32.6 ± 9.6	29.4 ± 6.5	0.318
AHI, events/h	8.6 ± 3.4	1.9 ± 1.3	**0.000**
AHI REM, events/h	18.3 ± 11.2	5.2 ± 4.7	**0.000**
AHI NREM, events/h	6.0 ± 3.8	1.2 ± 1.0	**0.000**
ODI, events/h	8.4 ± 3.6	1.9 ± 1.1	**0.000**
ODI REM, events/h	25.5 ± 30.1	5.5 ± 4.8	**0.000**
ODI NREM, events/h	5.8 ± 4.0	1.2 ± 0.9	**0.000**
Arousal index, events/h	22.4 ± 9.8	16.4 ± 10.1	**0.039**
ESS score	6.8 ± 4.3	6.7 ± 3.6	0.888
TST, min	302.5 ± 35.5	298.5 ± 48.4	0.621
Sleep efficiency, %	76.3 ± 9.3	74.5 ± 10.9	0.539
Sleep latency, min	61.5 ± 32.9	72.7 ± 39.5	0.520
WASO, min	34.2 ± 17.9	33.6 ± 33.4	0.176
Sleep stages, % TST			
N1	3.0 ± 1.9	3.1 ± 1.7	0.608
N2	49.4 ± 6.2	51.9 ± 8.7	0.261
N3	25.2 ± 7.4	25.1 ± 9.8	0.668
REM	19.8 ± 5.4	17.1 ± 6.9	0.134
Nocturnal mean SpO_2_, %	91.0 ± 2.8	92.8 ± 1.5	**0.025**
Nocturnal minimum SpO_2_, %	83.8 ± 3.6	89.1 ± 2.1	**0.000**
spO_2_ < 90%, % TST	30.6 ± 36.7	1.7 ± 4.4	**0.000**
spO2 < 90%, min	94.9 ± 115.6	5.0 ± 11.8	**0.000**
Nocturnal respiratory rate, breaths/min	21.0 ± 4.3	21.6 ± 3.8	0.613
Nocturnal heart rate, beats/min	71.8 ± 12.9	64.4 ± 10.2	**0.049**
BMI, kg/m^2^	21.9 ± 3.7	21.2 ± 3.0	0.737
FEV_1_, L	2.0 ± 0.8	2.1 ± 0.8	0.608
FEV_1_, % predicted	49.1 ± 14.8	54.2 ± 14.7	0.233
FVC, L	3.3 ± 1.1	3.4 ± 1.0	0.920
FVC, % predicted	66.4 ± 17.9	71.7 ± 14.9	0.252
RV, L	3.2 ± 0.9	2.9 ± 0.8	0.303
RV, % predicted	179.9 ± 37.7	180.0 ± 45.1	0.996
Awake SpO_2_, %	95.4 ± 1.8	96.0 ± 1.5	0.136
Awake pO_2_, mmHg	75.7 ± 8.9	80.9 ± 8.4	**0.048**
Awake pCO_2_, mmHg	39.5 ± 2.7	38.5 ± 2.8	0.256
Awake pH	7.42 ± 0.02	7.40 ± 0.03	**0.023**
Awake HCO_3_^−^, mmol/L	25.6 ± 1.6	24.1 ± 1.7	**0.003**

Values are mean ± standard deviation

AHI, apnea-hypopnea index; ESS, Epworth Sleepiness Scale; BMI, body mass index; HCO_3_^−^, bicarbonate; NREM, non-rapid eye movement sleep; pCO_2_, partial pressure of carbon dioxide pressure; pO_2_, partial pressure of oxygen; ppFEV_1_, percent predicted forced expiratory volume in 1 s; ppFVC, percent predicted forced vital capacity; ODI, oxygen desaturation index; OSA, obstructive sleep apnea; REM, rapid eye movement sleep; RV, residual volume; SpO_2_, oxygen saturation; TST, total sleep time; WASO, wake after sleep onset

Bold values denote statistical significance at the *p* < 0.05 level

#### Profiles of pwCF with and without nocturnal hypoxemia

Thirteen (25%) pwCF had relevant nocturnal hypoxemia with SpO2 < 90% for ≥ 5% of TST with a nadir of at least 85%. Those with versus without nocturnal hypoxemia had significantly lower ppFEV_1_, ppFVC and markers of awake oxygenation, and significantly higher AHI, AHI REM and AHI NREM (Table 4). There were no significant differences between patient groups in RV, age, BMI, ESS score, nocturnal respiratory and heart rates, awake pCO_2_, and sleep quality and architecture (Table 4). Only one of the thirteen patients with nocturnal hypoxemia had an ESS score > 10 (Table 4).

**Table 4:** Comparison of clinical characteristics and polysomnographic data in people with cystic fibrosis with and without nocturnal hypoxemia

	Hypoxemia (*n* = 13)	No hypoxemia (*n* = 39)	p value
Age, years	34.4 ± 10.2	29.4 ± 6.8	0.120
AHI, events/h	8.8 ± 3.8	3.2 ± 3.1	**0.000**
AHI REM, events/h	20.3 ± 11.8	7.2 ± 7.3	**0.001**
AHI NREM, events/h	5.8 ± 4.3	2.3 ± 2.6	**0.000**
ODI, events/h	9.0 ± 4.0	3.1 ± 2.9	**0.000**
ODI REM, events/h	21.5 ± 10.6	7.4 ± 7.3	**0.000**
ODI NREM, events/h	6.1 ± 4.8	2.1 ± 2.4	**0.000**
Arousal index, events/h	20.9 ± 9.8	18.1 ± 10.5	0.409
ESS score	5.9 ± 3.0	6.8 ± 4.1	0.400
TST, min	308.3 ± 21.5	297.3 ± 48.4	0.575
Sleep efficiency, %	78.0 ± 7.6	74.4 ± 10.9	0.279
Sleep latency, min	59.9 ± 27.2	70.9 ± 39.7	0.634
WASO, min	36.8 ± 17.0	32.8 ± 30.9	0.139
Sleep stages, % TST			
N1	3.1 ± 1.9	3.1 ± 1.7	0.916
N2	48.2 ± 5.3	51.7 ± 8.4	0.159
N3	26.9 ± 6.3	24.6 ± 9.5	0.148
REM	19.1 ± 5.7	17.9 ± 6.7	0.565
Nocturnal mean SpO_2_, %	89.6 ± 2.6	92.9 ± 1.5	**0.000**
Nocturnal minimum SpO_2_, %	81.4 ± 2.2	88.8 ± 2.0	**0.000**
Nocturnal respiratory rate, breaths/min	22.7 ± 4.6	20.1 ± 3.7	0.172
Nocturnal heart rate, beats/min	72.9 ± 13.0	65.5 ± 11.0	0.064
BMI, kg/m^2^	21.8 ± 3.9	21.4 ± 3.1	0.983
FEV_1_, L	1.7 ± 0.6	2.2 ± 0.8	**0.014**
FEV_1_, % predicted	43.5 ± 13.0	55.0 ± 14.4	**0.014**
FVC, L	2.9 ± 0.1	3.5 ± 1.0	**0.046**
FVC, % predicted	60.8 ± 16.1	72.5 ± 15.2	**0.023**
RV, L	3.4 ± 1.0	2.9 ± 0.8	0.068
RV, % predicted	196.9 ± 43.2	174.3 ± 40.5	0.092
Awake SpO_2_, %	95.0 ± 1.8	96.1 ± 1.6	**0.021**
Awake pO_2_, mmHg	72.5 ± 8.4	80.8 ± 8.2	**0.003**
Awake pCO_2_, mmHg	40.2 ± 2.5	38.4 ± 2.7	0.051
Awake pH	7.42 ± 0.02	7.41 ± 0.03	0.175
Awake HCO_3_^−^, mmol/L	25.7 ± 1.7	24.4 ± 1.7	**0.017**

Values are mean ± standard deviation

AHI, apnea-hypopnea index; ESS, Epworth Sleepiness Scale; BMI, body mass index; FEV_1_, forced expiratory volume in 1 s; FVC, forced vital capacity; HCO_3_^−^, bicarbonate; NREM, non-rapid eye movement sleep; ODI, oxygen desaturation index; pCO_2_, partial pressure of carbon dioxide pressure; pO_2_, partial pressure of oxygen; RDI, respiratory disturbance index; REM, rapid eye movement sleep; RV, residual volume; SpO_2_, oxygen saturation; TST, total sleep time; WASO, wake after sleep onset

Bold values denote statistical significance at the *p* < 0.05 level

## Profiles of pwCF with and without daytime sleepiness

The proportion of adult pwCF with an ESS score > 10, indicating EDS, was 15% (Table 5). The only significant difference between pwCF with and without EDS was for BMI (p = 0.040) (Table 5).

**Table 5:** Comparison of clinical characteristics and polysomnographic data in people with cystic fibrosis with or without daytime sleepiness (Epworth Sleepiness Scale score > 10 versus ≤ 10)

	ESS score ≥ 10 (*n* = 8)	ESS score < 10 (*n* = 44)	p value
Age, years	28.9 ± 4.8	31.0 ± 8.4	0.718
AHI, events/h	4.1 ± 3.2	4.7 ± 4.2	0.833
AHI REM, events/h	7.7 ± 5.4	11.0 ± 10.9	0.970
AHI NREM, events/h	3.2 ± 3.5	3.2 ± 3.5	0.872
ODI, events/h	3.7 ± 2.7	4.7 ± 4.2	0.891
ODI REM, events/h	7.6 ± 5.4	14.4 ± 23.7	0.694
ODI NREM, events/h	2.7 ± 2.8	3.1 ± 3.6	0.911
Arousal index, events/h	22.4 ± 8.2	18.1 ± 10.6	0.292
TST, min	313.4 ± 29.0	297.7 ± 45.3	0.276
Sleep efficiency, %	79.2 ± 8.4	74.6 ± 10.4	0.245
Sleep latency, min	51.3 ± 20.7	71.2 ± 38.7	0.155
WASO, min	30.8 ± 21.5	34.4 ± 29.2	0.813
Sleep stages, % TST			
N1	2.8 ± 0.9	3.1 ± 1.9	0.970
N2	54.4 ± 5.4	50.2 ± 8.1	0.167
N3	21.9 ± 4.6	25.8 ± 9.3	0.375
REM	18.6 ± 5.6	18.1 ± 6.6	0.833
Nocturnal mean SpO_2_, %	92.4 ± 1.9	92.0 ± 2.4	0.718
Nocturnal minimum SpO_2_, %	88.4 ± 3.0	86.7 ± 3.9	0.187
SpO_2_ < 90%, % TST	7.9 ± 21.0	14.4 ± 28.3	0.133
SpO_2_ < 90, min	23.0 ± 60.2	44.6 ± 89.5	0.097
Nocturnal respiratory rate, breaths/min	20.5 ± 3.2	21.6 ± 4.1	0.478
Nocturnal heart rate, beats/min	60.9 ± 7.0	68.5 ± 12.2	0.108
BMI, kg/m^2^	23.4 ± 2.6	21.1 ± 3.1	**0.040**
FEV_1_, L	2.1 ± 0.8	2.1 ± 0.8	0.990
FEV_1_, % predicted	52.0 ± 15.3	52.2 ± 14.9	0.978
FVC, L	3.4 ± 1.2	3.4 ± 1.0	0.897
FVC, % predicted	70.9 ± 18.8	69.3 ± 15.9	0.803
RV, L	2.7 ± 0.6	3.1 ± 0.9	0.329
RV, % predicted	172.8 ± 40.5	181.3 ± 42.5	0.603
Awake SpO_2_, %	96.4 ± 1.1	95.7 ± 1.7	0.276
Awake pO_2_, mmHg	78.0 ± 6.1	78.9 ± 9.4	0.789
Awake pCO_2_, mmHg	38.4 ± 2.9	29.0 ± 2.8	0.591
Awake pH	7.4 ± 0.02	7.4 ± 0.03	0.335
Awake HCO_3_^−^, mmol/L	25.1 ± 1.3	24.6 ± 1.9	0.543

Values are mean ± standard deviation

AHI, apnea-hypopnea index; ESS, Epworth Sleepiness Scale; BMI, body mass index; FEV_1_, forced expiratory volume in 1 s; FVC, forced vital capacity; HCO_3_^−^, bicarbonate; NREM, non-rapid eye movement sleep; ODI, oxygen desaturation index; pCO_2_, partial pressure of carbon dioxide pressure; pO_2_, partial pressure of oxygen; RDI, respiratory disturbance index; REM, rapid eye movement sleep; RV, residual volume; SpO_2_, oxygen saturation; TST, total sleep time; WASO, wake after sleep onset

Bold values denote statistical significance at the *p* < 0.05 level

### Prediction of OSA and sleep hypoxemia

**Fig. 1:**
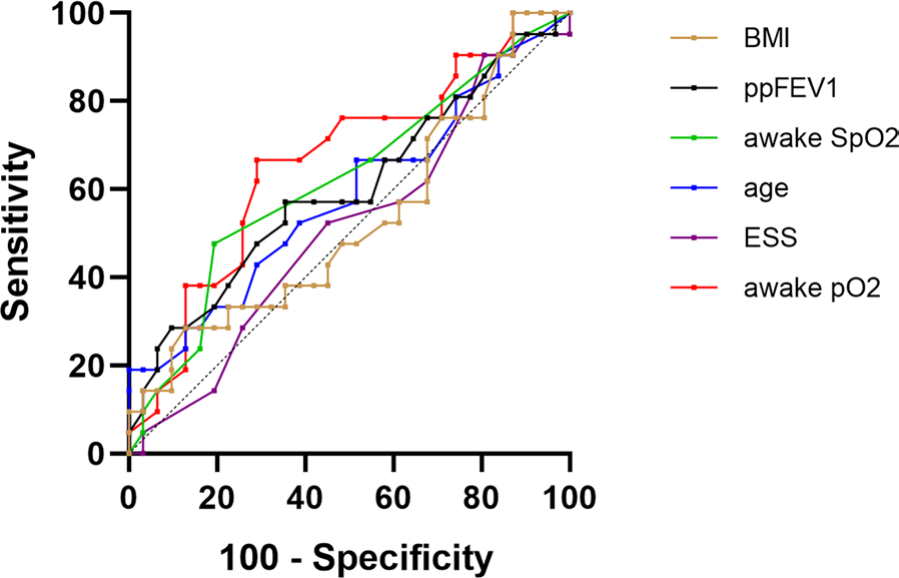
Receiver operating characteristic (ROC) curves for prediction of obstructive sleep apnea using percent predicted forced expiratory volume in 1 s (ppFEV_1_), body mass index (BMI), age, awake oxygen saturation (SpO_2_), awake partial pressure of oxygen (pO_2_) and Epworth Sleepiness Scale (ESS) score

**Fig. 2:**
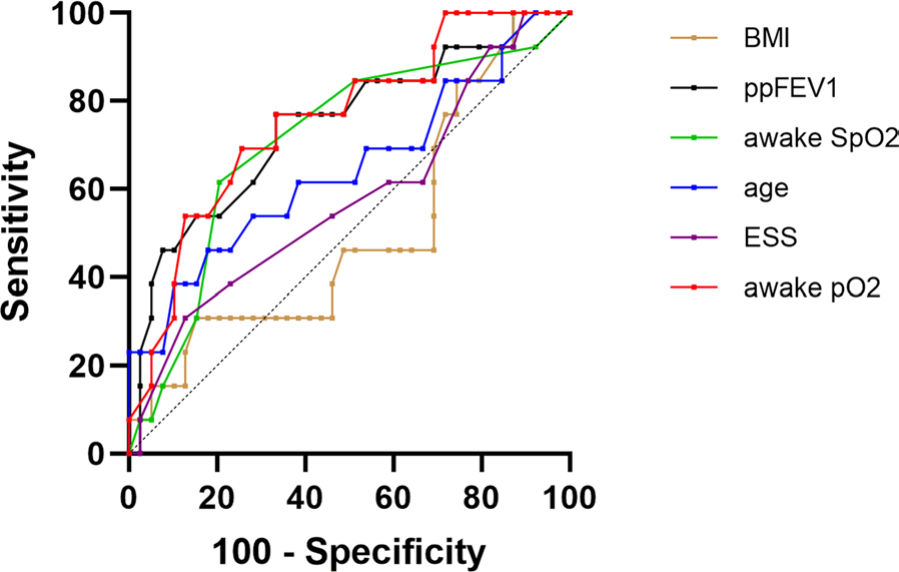
Receiver operating characteristic (ROC) curves for prediction of nocturnal hypoxemia using percent predicted forced expiratory volume in 1 s (ppFEV_1_), body mass index (BMI), age, awake oxygen saturation (SpO_2_), awake partial pressure of oxygen (pO_2_) and Epworth Sleepiness Scale (ESS) score

ROC curves and ROC analysis (see Table 6) were performed to compare the accuracy of ppFEV_1_, age, BMI, ESS score, awake pO_2_ and SpO_2_ to predict OSA (see Fig. 1) or nocturnal hypoxemia (see Fig. 2).

**Table 6:** Receiver operator characteristics (ROC) analysis for predicting obstructive sleep apnea and nocturnal hypoxemia

	OSA					Nocturnal hypoxemia				
	AUC	p	Cut-off value	Sensitivity (95% CI)	Specificity (95% CI)	AUC	p	Cut-off value	Sensitivity (95% CI)	Specificity (95% CI)
BMI, kg/m^2^	0.53	0.737	> 21	47.6 (28.3–67.6)	51.6 (34.8–68.0)	0.50	0.983	> 21	46.2 (23.2–70.9)	51.3 (36.2–66.1)
FEV_1_, % predicted	0.59	0.225	< 49.5	57.1 (36.6–75.5)	64.5 (47.0-79.9)	0.74	**0.009**	< 49.5	76.9 (49.7–91.8)	66.7 (51.0-79.4)
Awake pO_2_, mmHg	0.66	**0.048**	< 78.5	66.7 (45.4–82.8)	71.0 (53.4–84.0)	0.76	**0.006**	< 78.5	76.9 (49.7–91.8)	66.7 (51.0-79.4)
Age, years	0.58	0.318	> 28.5	66.7 (45.4–82.1)	48.4 (32.0-65.2)	0.65	0.120	> 30.5	61.5 (35.5–82.3)	61.5 (46.0-75.1)
ESS score	0.51	0.888	< 5.5	52.4 (32.4–71.7)	54.8 (37.8–70.9)	0.58	0.404	< 6.5	61.5 (35.5–82.3)	41.0 (27.1–56.6)
Awake SpO_2_, %	0.62	0.148	< 96.5	66.7 (45.4–82.8)	45.2 (29.2–62.2)	0.71	**0.025**	< 95.5	61.5 (35.5–82.3)	79.5 (64.5–89.2)

AUC, area under the ROC curve; BMI, body mass index; CI, confidence interval; ESS, Epworth Sleepiness Scale; FEV_1_, forced expiratory volume in 1 s; OSA, obstructive sleep apnea; pO_2_, partial pressure of oxygen; SpO_2_, oxygen saturation

Bold values denote statistical significance at the *p* < 0.05 level

The only significant predictor of OSA was awake pO_2_ (AUC 0.66, p = 0.048) (Fig. 1; Table 6). The best predictors of nocturnal hypoxemia in this population were pFEV_1_, awake pO_2_ and SpO_2_ (Fig. 2; Table 6). BMI, age and ESS score were not good predictors of nocturnal hypoxemia (Fig. 2; Table 6).

The pO_2_ cut-off value with the greatest ROC for predicting AHI was < 78.5 mmHg with a sensitivity of 67% and specificity of 71%. For predicting nocturnal hypoxemia, cut-off values were < 49.5 for ppFEV_1_ (sensitivity 76.9%, specificity 66.7%), < 78.5 mmHg for awake pO_2_ (sensitivity 76.9%, specificity 66.7%), and < 95.5% for awake SpO_2_ (sensitivity 61.5%, specificity 79.5%).

## Discussion

To the best of our knowledge, this is one of the largest sleep studies evaluating adult pwCF. The main finding of our study is a high prevalence of OSA and nocturnal hypoxemia in this population, and that ability of clinical markers to predict OSA is very limited, whereas the incidence of nocturnal hypoxemia is dependent on lung function parameters and awake oxygenation markers (SpO_2_ and pO_2_). EDS with an ESS score > 10 was also a common finding, SDB did not significantly influence the occurrence of EDS.

Although the AHI of the entire population in this study was normal, consistent with existing literature [13, 21, 24, 32], there was a high prevalence of OSA (AHI ≥ 5/h). Comparing our data with the available literature is challenging, because study designs and patient selection are heterogenous with respect to age, lung function, BMI and sample size, and PSG studies examining an exclusively adult CF population are rare [7, 33]. Published data regarding the prevalence of OSA in adult pwCF report a prevalence of 3.2–3.9% [13, 32], substantially lower than the 40% in our study. However, our data are more in line with adult patients with non-CF-bronchiectasis, which showed a similarly high prevalence of OSA [34, 35]. Due to the diversity of studies and patient characteristics, it must be assumed that the prevalence of OSA is often underestimated, and further studies are needed to confirm our findings. This assumption is supported by the fact that the CF population is aging due to improved treatment options and obesity is an increasing problem [4, 36].

Outside the field of CF, the co-existence of OSA and chronic obstructive airflow limitation (OSA-COPD overlap syndrome) has received increasing attention in recent years. The prevalence of OSA-COPD overlap is 10–65%, depending on study design and patient selection [6]. There is growing evidence that patients with OSA-COPD overlap experience more pulmonary exacerbations and have a higher mortality than patients with COPD alone [37, 38]. In addition, patients with OSA-COPD and concomitant bronchiectasis had higher AHI and lower nocturnal oxygen levels than patients with OSA or COPD alone [39]. Comparable data do not exist for pwCF. Whether this knowledge can be transferred to pwCF should be investigated in further longitudinal studies.

Another poorly discussed question regarding OSA in adult pwCF is the role of the upper airways. CF results in impaired mucociliary clearance in the upper respiratory tract as well as the lungs, which may lead to the development of chronic rhinosinusitis (CRS) [40]. CRS may narrow the upper airways and impair breathing through the nose especially during sleep. Combining questionnaires and standard otolaryngology examination, alterations of the upper airways in children and adolescents with CF due to nasal polyposis and chronic infection are related to the development of OSA syndrome [41]. However, radiologic staging of upper airway patency is poorly standardized. In a study by Veronezi et al. [37], the Lund-Mackay score was used to assess upper airways in adolescents and young adults with CF. In their analysis, there was no association between the involvement of upper airways and AHI. Corresponding data for adult pwCF are lacking. The presence of CRS seems does not affect the AHI [42], but patients with CRS objectively report poor sleep quality [43]. Prevalence data on the co-occurrence of CRS and OSA in non-CF patients vary widely, between 15% and 64.7% [44, 45].

Our ROC analysis showed that classical clinical markers such as ppFEV_1_, BMI, age, and awake pO_2_ and SpO_2_ were poor predictors of the AHI. This is another area where data in adult pwCF are rare. In a significantly younger and less affected CF patient group, Veronezi et al. showed that nutritional status, awake SpO_2_ and daytime sleepiness were closely associated with the presence of OSA [46]. We were not able to confirm these findings in our analysis because we found only a weak association between awake pO_2_ and the presence of OSA. All other factors, including ESS score, BMI, ppFEV_1_ and awake SpO_2_ were not significantly associated with the presence of OSA in adult pwCF in the ROC analysis.

Nocturnal hypoxia is a common finding in pwCF. As with in other sleep studies in pwCF [16, 17, 47, 48], our work also showed a correlation between the severity of lung involvement in CF and the occurrence of nocturnal hypoxia. Detection and correction of nocturnal hypoxia can have a major impact on disease progression and the person’s well-being. Chronic nocturnal hypoxia can cause of sleep disturbance, impaired glucose regulation, decreased quality of life, development of pulmonary hypertension, impaired neurocognitive function, and daytime sleepiness [24, 49, 50]. Therefore, early detection of nocturnal hypoxemia is important. Our data support the findings by others that l awake oxygenation (SpO_2_ and pO_2_) seem to be the most important clinical predictors of nocturnal hypoxemia in adult pwCF, whereas ppFEV_1_ has been shown to be a good predictor of nocturnal hypoxemia in children with CF [13, 15, 48, 51].

Most current data regarding sleep quality and sleep architecture in pwCF come from studies in children and adolescents. Consistent with existing literature in adult pwCF [22, 24, 52, 53], we confirmed that these patients have reduced sleep quality (total sleep time, sleep efficiency) and increased sleep latency and wake after sleep onset. There are multiple potential contributors to impaired subjective and objective sleep in pwCF, including nocturnal coughing, pain, chronic rhinosinusitis, CF-related diabetes, and PEG-feeding [54]. However, sleep architecture (N1, N2, N3 and REM stages) was preserved despite reduced sleep quality. These findings are consistent with data from other studies in children and adult pwCF [13, 19, 23, 53].

Although EDS is one of the main symptoms of OSA, the relationship between AHI and EDS in individuals without CF is inconsistent [55]. This is in line with our findings showing that the overall ESS score was in the normal range even though the prevalence of OSA and nocturnal hypoxia was high. In a study by Bouka et al., clinically stable adult pwCF showed elevated ESS scores compared with healthy individuals, indicating a higher level of daytime sleepiness [20]. In their study, nearly 20% of the examined adult pwCF had an ESS score of > 10, similar to the 15% in our study. In addition, both our study and the one by Bouka et al. reported that the overall ESS score was in the normal range.

In our study, there were no differences between patients with high vs. low ESS scores in terms of SDB, sleep architecture, sleep quality or respiratory markers. This suggests that there must be other factors that influence daytime sleepiness besides sleep and respiratory markers. Depression and anxiety are well known comorbidities in pwCF [56], and these can impact on sleep quality [24, 54]. We can only speculate that there is an association between daytime sleepiness and depression/anxiety in pwCF. However, data from individuals without CF show that the presence of daytime sleepiness is more associated with depression than with SDB [55].

Our study has a number of strengths, including a large number of patients with a wide range of disease severity, but there are also some limitations to note. The main limitation is that we do not have data on nocturnal hypoventilation to provide a complete picture of SDB in adult pwCF. With progression of the disease and a further decline in lung function, nocturnal hypoventilation with consecutive hypercapnia is detectable with possible need for noninvasive ventilation [57]. In this context, nocturnal carbon dioxide levels, preferably measured transcutaneously, are part of a comprehensive sleep assessment. Furthermore, as we know from numerous other studies, there is an association between sleep and HRQoL [49, 54]. However, we did not have HRQoL data for our patients and were therefore unable to investigate associations between HRQoL and objective sleep parameters.

## Conclusion

In summary, our data show a high prevalence of OSA, nocturnal hypoxemia and EDS in adult pwCF. OSA and nocturnal hypoxemia were mainly detected in REM sleep. There was no difference between patients with and without OSA (AHI ≥ 5/h) did not differ significantly with respect to age, lung function and weight, but had significantly lower awake oxygen levels. The occurrence and duration of nocturnal hypoxemia were dependent on lung function and awake oxygenation. None of the clinical markers assessed was a significant predictor of OSA, whereas ppFEV_1_, awake SpO_2_ and pO_2_ were good predictors of the occurrence of nocturnal hypoxemia. Neither the presence of OSA nor nocturnal hypoxemia had any influence on the ESS score. Based on our data, we suggest regular PSG screening to detect OSA and nocturnal hypoxemia in adult pwCF, regardless of disease severity. This could help to prevent medical deterioration due to undetected SDB in pwCF.

## Data Availability

The data used to support the current findings are available from the corresponding author upon request.

## Abbreviations

AHI: Apnea-hypopnea index
AUC: Area under the curve
BMI: Body mass index
CF: Cystic fibrosis
CFTR: Cystic fibrosis transmembrane conductance regulator
Cl^−^: Chloride
COPD: Chronic obstructive pulmonary disease
EDS: Excessive daytime sleepiness
ESS: Epworth Sleepiness Scale
FEV_1_: Forced expiratory volume in 1 s
FVC: Forced vital capacity
HCO_3_^−^: Bicarbonate
OSA: Obstructive sleep apnea
PFT: Pulmonary function testing
pCO_2_: Partial pressure of carbon dioxide
pO_2_: Partial pressure of oxygen
PSG: Polysomnography
ppFEV_1_: Percent predicted forced expiratory volume in 1 s
pwCF: People with cystic fibrosis
REM: Rapid eye movement
ROC: Receiver operator characteristics
RV: Residual volume
SDB: Sleep-disordered breathing
SpO_2_: Oxygen saturation
TST: Total sleep time
